# Neuropsychological markers of antidepressant action: a secondary analysis of the ANTLER randomised controlled trial

**DOI:** 10.1017/S0033291722003981

**Published:** 2023-10

**Authors:** Julia Rodriguez-Sanchez, Gemma Lewis, Francesca Solmi, Jessica K. Bone, Michael Moore, Nicola Wiles, Catherine J. Harmer, Larisa Duffy, Glyn Lewis

**Affiliations:** 1 Division of Biosciences, UCL, London, UK; 2 Division of Psychiatry, UCL, London, UK; 3 Department of Behavioural Science and Health, Institute of Epidemiology and Health Care, UCL, London, UK; 4 Primary Care Population Sciences and Medical Education, Faculty of Medicine, University of Southampton, Southampton, UK; 5 Centre for Academic Mental Health, Bristol Medical School, University of Bristol, Bristol, UK; 6 University Department of Psychiatry, Warneford Hospital, Oxford, UK; 7 Oxford Health NHS Foundation Trust, Warneford Hospital, Oxford, UK

**Keywords:** Antidepressants, cognitive neuropsychological model, depression, emotional processing, memory, recall

## Abstract

**Background:**

Antidepressants have been proposed to act via their influence on emotional processing. We investigated the effect of discontinuing maintenance antidepressant treatment on positive and negative self-referential recall and the association between self-referential recall and risk of relapse.

**Methods:**

The ANTLER trial was a large (*N* = 478) pragmatic double-blind trial investigating the clinical effectiveness of long-term antidepressant treatment for preventing relapse in primary care patients. Participants were randomised to continue their maintenance antidepressants or discontinue via a taper to placebo. We analysed memory for positive and negative personality descriptors, assessed at baseline, 12- and 52-week follow-up.

**Results:**

The recall task was completed by 437 participants. There was no evidence of an effect of discontinuation on self-referential recall at 12 [positive recall ratio 1.00, 95% CI (0.90–1.11), *p* = 0.93; negative recall ratio 1.00 (0.87–1.14), *p* = 0.87] or 52 weeks [positive recall ratio 1.03 (0.91–1.17), *p* = 0.62; negative recall ratio 1.00 (0.86–1.15), *p* = 0.96; ratios larger than one indicate higher recall in the discontinuation group], and no evidence of an association between recall at baseline or 12 weeks and later relapse [baseline, positive hazard ratio (HR) 1.02 (0.93–1.12), *p* = 0.74; negative HR 1.01 (0.90–1.13), *p* = 0.87; 12 weeks, positive HR 0.99 (0.89–1.09), *p* = 0.81; negative HR 0.98 (0.84–1.14), *p* = 0.78; ratios larger than one indicate a higher frequency of relapse in those with higher recall].

**Conclusions:**

We found no evidence that discontinuing long-term antidepressants altered self-referential recall or that self-referential recall was associated with risk of relapse. These findings suggest that self-referential recall is not a neuropsychological marker of antidepressant action.

## Introduction

Selective serotonin reuptake inhibitor (SSRI) antidepressants are often a first line treatment for depression (Kendrick et al., 2009). SSRIs are amongst the most commonly prescribed medications in high-income countries and have seen a dramatic and steady increase in prescriptions over the past three decades (NHS Digital, 2019). Although antidepressants are commonly used for treating acute depressive episodes, a large and increasing proportion of prescriptions are for maintenance treatment to prevent future relapses. Between 1993 and 2005, an estimated 90% of prescriptions were for maintenance treatment, with the amount of time spent on SSRIs rising from 0.73 years per 100-person years in 1995 to 4.94 in 2012 (McCrea, Sammon, Nazareth, & Petersen, 2016; Moore et al., 2009).

The cognitive neuropsychological model of antidepressant action proposes that depression is associated with biases towards negative and away from positive information, and that treatments for depression modulate such biases, for instance by biasing emotional processing towards positive stimuli (Clark, Chamberlain, & Sahakian, 2009; Harmer et al., 2009a; Roiser, Elliott, & Sahakian, 2012). In line with this model, there is evidence that antidepressants alter emotional processing (Harmer, Duman, & Cowen, 2017). Changes in emotional processing have been found to precede changes in mood and symptoms (Harmer, Goodwin, & Cowen, 2009b; Lewis et al., 2017; Tranter et al., 2009).

Emotional memory is a candidate neuropsychological marker of antidepressant drug action. Self-referential recall, which can be measured by asking participants to classify then recall positive and negative personality characteristics, has been used to investigate the effects of antidepressants. Several studies have found increased positive and/or decreased negative recall following administration of an antidepressant (Arnone, Horder, Cowen, & Harmer, 2009; Harmer, Shelley, Cowen, & Goodwin, 2004). However, most of the existing evidence comes from experimental studies in healthy volunteers, often with small sample sizes and acute or short-term interventions. Such studies have limited generalisability, and there have been inconsistent findings (Ahmed et al., 2021; Komulainen et al., 2018; Walsh et al., 2018).

The early effects of antidepressants on emotional memory have attracted considerable attention, but the impact of antidepressant discontinuation on these processes and their potential to guide treatment decisions has not been addressed. Emotional biases towards negative information have been proposed as a vulnerability factor for depression relapse, but this hypothesis has not been appropriately tested (Bouhuys, Geerts, & Gordijn, 1999). In this study, we investigated the effect of discontinuing maintenance antidepressants on positive and negative self-referential recall and examined the association between recall and subsequent risk of relapse.

Reduced serotonin availability has been shown to alter emotional processing in acute tryptophan depletion studies. Contrary to antidepressant administration, tryptophan depletion has been shown to decrease positive biases and increase negative biases in both healthy participants and remitted patients (Hayward, Goodwin, Cowen, & Harmer, 2005). Consistent with this, reduced serotonin availability upon antidepressant discontinuation could lead to decreased positive and increased negative stimuli processing. This should be particularly apparent in the first few weeks after discontinuation, when patients may experience withdrawal symptoms. In patients, such changes in emotional processing are generally associated with changes in mood (Smith, Fairburn, & Cowen, 1997; Van der Does, 2001), and could reflect an increased risk of relapse.

We therefore hypothesised (i) that recall of self-referential positive words would decrease and recall of self-referential negative words would increase in the discontinuation group, relative to maintenance antidepressant treatment, and (ii) that decreased recall of self-referential positive words and increased recall of self-referential negative words would be associated with subsequent risk of relapse.

## Methods

### Study design and participants

We analysed data from ANTLER, a large individually randomised (*N* = 478) pragmatic double-blind trial that investigated the clinical effectiveness of antidepressants for preventing relapse in primary care patients receiving long-term maintenance treatment (ANTLER; Duffy et al., 2019; Lewis et al., 2021). The ANTLER trial found evidence that discontinuing treatment increased the risk of relapse. However, over 40% of primary care patients who discontinued were able to do so without relapsing over the 12-month follow-up (Lewis et al., 2021).

The ANTLER trial was registered with Controlled Trials ISRCTN Registry (reference ISRCTN15969819). All procedures complied with the ethical standards of the relevant national and institutional committees on human experimentation and with the Helsinki Declaration of 1975, as revised in 2008.

Participants were recruited from 150 general practices across four research centres (in London, Bristol, Southampton and York), either via record search or in-consultation recruitment. Eligible individuals were primary care patients being treated for depression but who were well enough to consider stopping antidepressant medication. Patients were assessed for the following inclusion criteria: aged 18–74 years, at least two episodes of depression, antidepressant treatment for at least 9 months and currently taking citalopram 20 mg, sertraline 100 mg, fluoxetine 20 mg or mirtazapine 30 mg, and adherence to medication (defined using a five-item self-report measure of compliance).

Participants were excluded if they met any of the following criteria: internationally agreed (ICD-10) criteria for a depressive illness (assessed using the CIS-R), comorbid bipolar disorder, psychotic illness, dementia or a terminal illness, inability to complete self-administered questionnaires in English, contraindications for any of the prescribed medication, concurrent enrolment in another investigational medicinal product (IMP) trial, current or planned pregnancy or breastfeeding, use of monoamine oxidase inhibitors, and allergies to placebo excipients.

### Randomisation procedure

Eligible participants completed a baseline assessment and provided a written consent. They were then randomised 1:1 with a remote computer-generated code, either to remain on active antidepressant medication (citalopram 20 mg, sertraline 100 mg, fluoxetine 20 mg or mirtazapine 30 mg) or to take an identical placebo following a 1-month (for fluoxetine) or 2-month (for citalopram, sertraline and mirtazapine) tapering period. Randomisation was stratified by study centre, antidepressant medication and severity of depressive symptoms.

The active medication and placebo were encapsulated, with all capsules identical in dimensions and appearance. Trial participants, clinicians and all members of the research team were blinded to the treatment allocation (Duffy et al., 2019). Analyses for this study were performed with awareness of the random allocation.

### Measures

The duration of the trial was of 52 weeks. Follow-up assessments were carried out at 6, 12, 26, 39 and 52 weeks after randomisation (Duffy et al., 2019). Participants completed the word recall task at baseline and at 12 and 52 weeks after randomisation.

#### Word recall task

The word recall task tests memory of positive and negative information. Participants were first asked to classify 20 positive (e.g. cheerful) and 20 negative (e.g. hostile) personality characteristics by indicating whether they would ‘like’ or ‘dislike’ to hear someone describing them in this way. The words were presented in a random order for 500 ms. Positive and negative words were matched according to length, usage frequency and meaningfulness. This emotional categorisation task (ECAT) was immediately followed by a surprise test, in which participants were asked to recall as many personality descriptors as possible in 2 min. Participants would have known to expect the recall element at follow-ups, but this did not lead to a marked improvement in recall in previous studies (Ahmed et al., 2021; Lewis et al., 2017). The number of positive and negative words recalled accurately (hits) and falsely (false alarms) were recorded.

#### Additional measures

Relapse was measured retrospectively at 12, 26, 39 and 52 weeks using the retrospective Clinical Interview Schedule-Revised (rCIS-R; Duffy *et al*., 2021; Lewis, Pelosi, Araya, & Dunn, 1992). The rCIS-R comprises five sections (depressive mood, depressive ideas, concentration, sleep and fatigue) and asks participants about symptoms in the previous 12 weeks. To be identified as having relapsed, participants had to report either low mood or anhedonia lasting for at least two weeks, together with at least one additional symptom from depressive thoughts, fatigue, loss of concentration, or sleep disturbance.

Participants also completed the Patient Health Questionnaire (PHQ-9; Kroenke, Spitzer, & Williams, 2001) and the Generalised Anxiety Disorder Assessment (GAD-7; Spitzer, Kroenke, Williams, and Löwe, 2006) at all times, which measured depressive and anxiety symptoms, respectively. Withdrawal symptoms were assessed based on the Discontinuation-Emergent Signs and Symptoms (DESS; Rosenbaum, Fava, Hoog, Ascroft, and Krebs, 1998).

Additional clinical and sociodemographic measures, including age, sex, ethnicity, educational qualifications, and past medical history (such as physical illness contraindications and past psychiatric treatments), were obtained as part of the baseline assessment.

### Statistical analysis

Analyses were performed using Stata 16 (StataCorp, 2019). Descriptive statistics are presented for the overall sample included in the analysis and separately for each treatment group. We analysed performance in the initial classification stage of the word recall task (the ECAT) at baseline (online Supplement), to ensure that participants understood the task, before analysing performance in the recall element of the task.

#### Associations between treatment allocation and word recall

We first examined performance in the word recall task at all time points (baseline, 12 weeks, and 52 weeks) according to treatment allocation. Next, we investigated the effect of treatment allocation (maintenance antidepressant or discontinuation) on task performance. We tested whether treatment allocation (exposure) was associated with positive or negative hits (outcomes modelled in separate models) at 12 or 52 weeks after randomisation.

Negative binomial regression models were used because hits were count variables that were positively skewed. From these models, we report the ratio of hits for the discontinuation relative to maintenance groups. A ratio greater than 1 indicates that word recall was higher in the discontinuation than maintenance group.

We examined whether there was evidence that the influence of treatment allocation on hits differed according to word valence (positive *v.* negative). To do this, we reshaped the data, using total hits at 12 or 52 weeks as the outcome, with treatment allocation and word valence (positive *v.* negative) as exposures. We included an interaction term between treatment allocation and word valence to investigate whether the effect of treatment on recall varied according to word valence.

All models were adjusted for baseline positive and/or negative hits to account for recall ability. They are reported before and after adjustment for false alarms (positive and negative) and stratification variables (severity of depressive symptoms at baseline, assessed using the CIS-R, medication and study centre).

#### Associations between word recall and relapse

We examined associations between task performance (positive and negative hits) at baseline and risk of relapse using Cox Proportional Hazards modelling. In this analysis we were treating the study as though it were a cohort study and adjusted for randomised treatment. The outcome for these analyses was time to relapse. We included baseline positive hits (adjusted for baseline negative hits) and baseline negative hits (adjusted for baseline positive hits) as exposures in two separate models. We report the hazard ratio estimates associated with positive and negative baseline hits.

All models were adjusted for positive and negative false alarms, treatment allocation, medication (citalopram, fluoxetine, sertraline or mirtazapine) and potential confounders including baseline depression and anxiety symptom severity (evaluated using the CIS-R, PHQ-9 and GAD-7), number of previous episodes of depression, duration of antidepressant treatment prior to randomisation, age, sex, and education.

#### Sensitivity analyses

In sensitivity analyses, we tested whether findings were altered by limiting the sample to (i) participants who adhered to study medication or (ii) those who performed well on the ECAT (80% accuracy and above; Ahmed et al., 2021). We also investigated whether adjusting for baseline variables associated with missingness in our outcomes altered our findings. Further analyses assessed potential treatment effect modification by baseline positive and negative hits (online Supplement).

## Results

### Descriptive statistics

A total of 478 participants were randomised, 238 to maintenance antidepressant treatment and 240 to treatment discontinuation. Forty-one participants (13 from the maintenance group and 28 from the discontinuation group) did not complete the recall task at 12 and 52 weeks. This left 437 participants for analyses: 225 in the maintenance group and 212 in the discontinuation group.

Completion of the recall task differed by treatment allocation and was lower in the discontinuation (88%) than maintenance group (95%). Study site and age were also associated with task completion at 52 weeks, with missing data more common for younger participants and those recruited from Bristol.

Demographic and clinical characteristics of the sample are presented in Table 1. Overall, 54% (235) of participants were aged 55–74, 74% (323) were female, and 95% (413) were white; 42% (184) of participants were recruited from London sites, 22% (96) from Bristol, 20% (88) from Southampton, and 16% (70) from York; 47% (205) of participants were taking citalopram, 33% (146) were taking fluoxetine, 16% (70) were taking sertraline and less than 4% (16) were taking mirtazapine.

**Table 1.** Baseline demographic and clinical characteristics by randomised group for the sample who completed the emotional processing task at 12 and/or 52 weeks and for the whole trial population

	Maintenance (*n* = 225)	Discontinuation (*n* = 212)	Total sample (*n* = 437)	Trial population (*n* = 478)
	*n*(%)			
Age				
18–34	22 (9.8)	13 (6.1)	35 (8.0)	39 (8.2)
35–54	81 (36.0)	86 (40.6)	167 (38.2)	186 (38.9)
55–74	122 (54.2)	113 (53.3)	235 (53.8)	253 (52.9)
Female	160 (71.1)	163 (76.9)	323 (73.9)	350 (73.2)
Ethnicity				
White	209 (92.9)	204 (96.2)	413 (94.5)	449 (93.9)
Ethnic minority	16 (7.1)	8 (3.8)	24 (5.5)	29 (6.1)
Marital status				
Married	139 (61.8)	142 (67.9)	281 (64.3)	307 (64.2)
Single	33 (14.7)	26 (12.3)	59 (13.5)	61 (12.8)
Separated or divorced	36 (16.0)	28 (13.2)	64 (14.7)	72 (15.1)
Widowed	17 (7.6)	16 (7.6)	33 (7.6)	38 (8.0)
Employment status				
Employed	133 (59.1)	135 (63.7)	268 (61.3)	292 (61.1)
Retired	67 (29.8)	58 (27.4)	125 (28.6)	139 (29.1)
Other	25 (11.1)	19 (9.0)	44 (10.1)	47 (9.8)
Educational qualification				
Diploma or higher	118 (52.4)	132 (62.3)	250 (57.2)	269 (56.3)
A-level or equivalent	33 (14.7)	23 (10.9)	56 (12.8)	58 (12.1)
GCSE or equivalent	50 (22.2)	38 (17.9)	88 (20.1)	99 (20.7)
No formal qualification	23 (10.2)	18 (8.5)	41 (9.4)	46 (9.6)
Site				
London	97 (43.1)	87 (41.0)	184 (42.1)	199 (41.6)
Bristol	47 (20.9)	49 (23.1)	96 (22.0)	102 (21.3)
Southampton	44 (19.6)	43 (20.3)	87 (19.9)	96 (20.1)
York	37 (16.4)	33 (15.6)	70 (16.0)	81 (17.0)
Antidepressant				
Sertraline	38 (16.9)	32 (15.1)	70 (16.0)	78 (16.3)
Citalopram	106 (47.1)	99 (46.7)	205 (46.9)	223 (46.7)
Fluoxetine	73 (32.4)	73 (34.4)	146 (33.4)	160 (33.5)
Mirtazapine	8 (3.6)	8 (3.8)	16 (3.7)	17 (3.6)
CIS-R score above median	109 (48.4)	96 (45.3)	205 (46.9)	227 (47.5)
3 or more previous episodes of depression	184 (81.8)	165 (77.8)	349 (79.9)	376 (78.7)
Time taking antidepressants				
9 months to a year	12 (5.3)	13 (6.1)	25 (5.7)	30 (6.3)
1 to 2 years	53 (23.6)	48 (22.6)	101 (23.1)	109 (22.8)
3 to 5 years	75 (33.3)	70 (33.0)	145 (33.2)	160 (33.5)
6 to 10 years	45 (20.0)	43 (20.3)	88 (20.1)	96 (20.1)
11 or more years	40 (17.8)	37 (17.5)	77 (17.6)	82 (17.2)
Mean (S.D.)				
Age (years) that first became aware of having depression	32.9 (15.9)	31.5 (13.9)	32.3 (15.0)	32.4 (15.2)
CIS-R depressive symptoms	3.20 (2.9)	2.79 (2.8)	3.00 (2.9)	3.05 (2.9)
CIS-R anxiety symptoms	1.50 (2.0)	1.29 (1.9)	1.40 (1.9)	1.43 (2.0)
PHQ-9 total score	3.81 (3.5)	3.84 (3.6)	3.83 (3.5)	3.83 (3.5)
GAD-7 total score	3.19 (3.0)	2.79 (3.0)	3.00 (3.0)	3.00 (3.0)

Note. CIS-R, Revised Clinical Interview Schedule; PHQ-9, Patient Health Questionnaire 9-item version; GAD-7, Generalised Anxiety Disorder 7-item version; S.D., standard deviation. Missing data for *n* = 1 participant in the maintenance group on highest educational qualification and *n* = 1 participant in the discontinuation group on highest educational qualification, time taking antidepressants and age that first became aware of having depression.

The mean scores for depressive and anxiety symptoms, as assessed by the PHQ-9 and GAD-7, were 3.83 [standard deviation (s.d.) = 3.5] and 3.00 (s.d. = 3.0), respectively. On the CIS-R, mean depressive and anxiety symptom scores were 3.00 (s.d. = 2.9) and 1.40 (s.d. = 1.9). The two groups were well-balanced at baseline (Table 1).

Performance in the ECAT was good at baseline (median per cent correct = 92.5%, Q1 – Q3 = 87.5–97.5), with no differences across groups. However, 14% of participants did not understand some of the words (scored below 80% at baseline), and 4% performed worse than chance (online Supplement).

### Antidepressant discontinuation and self-referential recall

Participants in both treatment groups made more positive than negative hits and false alarms at all time points. Positive hits and false alarms increased from baseline to 12 weeks and decreased at 52 weeks. Negative false alarms decreased from baseline to 12 and 52 weeks, whilst negative hits did not change over time (Table 2).

**Table 2.** Positive and negative word recall (hits and false alarms) according to treatment allocation

	Maintenance		Discontinuation	
	*n*	Mean (s.d.)	*n*	Mean (s.d.)
Positive hits				
Baseline	224	3.00 (1.71)	211	3.22 (1.78)
12 weeks	221	3.13 (1.97)	212	3.24 (2.23)
52 weeks	203	2.55 (1.59)	172	2.72 (1.84)
Negative hits				
Baseline	224	1.87 (1.43)	211	2.03 (1.38)
12 weeks	221	1.99 (1.43)	212	2.08 (1.63)
52 weeks	203	1.99 (1.59)	172	2.10 (1.62)
Positive false alarms				
Baseline	224	1.89 (1.71)	211	1.87 (1.70)
12 weeks	221	2.11 (1.85)	212	1.92 (1.75)
52 weeks	203	1.85 (1.67)	172	1.90 (1.77)
Negative false alarms				
Baseline	224	1.12 (1.25)	211	1.11 (1.40)
12 weeks	221	0.98 (1.25)	212	0.96 (1.09)
52 weeks	203	1.02 (1.28)	172	0.93 (1.08)

Note. s.d., standard deviation. Hits were words accurately recalled from the word categorisation task. False alarms were words not presented in the word categorisation task and falsely recalled.

There was no evidence of an association between treatment allocation and positive hits at 12 weeks (unadjusted hits ratio = 1.03, CI 0.91–1.17, *p* = 0.59; adjusted hits ratio = 1.00, CI 0.90–1.11, *p* = 0.93) or 52 weeks (unadjusted hits ratio = 1.06, CI 0.93–1.21, *p* = 0.37; adjusted hits ratio = 1.03, CI 0.91–1.17, *p* = 0.62) after randomisation (Table 3). There was also no evidence of an association between treatment allocation and negative hits at 12 weeks (unadjusted hits ratio = 1.05, CI 0.91–1.20, *p* = 0.52; adjusted hits ratio = 1.00, CI 0.87–1.14, *p* = 0.87) or 52 weeks (unadjusted hits ratio = 1.05, CI 0.90–1.24, *p* = 0.52; adjusted hits ratio = 1.00, CI 0.86–1.15, *p* = 0.96).

**Table 3.** Ratio of positive or negative hits in the antidepressant discontinuation group, relative to long-term maintenance treatment, 12 and 52 weeks after randomisation

Positive hits				Negative hits			
Model	*n*	Hits ratio (95% CI)	*p* value	Model	*n*	Hits ratio (95% CI)	*p* value
12 weeks							
Unadjusted	433	1.03 (0.91–1.17)	0.59	Unadjusted	433	1.05 (0.91–1.20)	0.52
Adjusted^a^	430	1.00 (0.90–1.11)	0.93	Adjusted^a^	430	1.00 (0.87–1.14)	0.87
52 weeks							
Unadjusted	375	1.06 (0.93–1.21)	0.37	Unadjusted	375	1.05 (0.90–1.24)	0.52
Adjusted^a^	368	1.03 (0.91–1.17)	0.62	Adjusted^a^	368	1.00 (0.86–1.15)	0.96

Note. CI, confidence interval.

a Positive hits adjusted for negative hits, baseline positive hits, positive and negative false alarms, and stratification variables (symptom severity at baseline, assessed using the CIS-R, medication and study centre). Negative hits adjusted for positive hits, baseline negative hits, positive and negative false alarms, and stratification variables. Results unaltered after adjusting for predictors of missingness.

Next, we investigated the effect of treatment on recall for positive *v.* negative hits. There was no evidence for an interaction between treatment and word valence at either time point (unadjusted hits at 12 weeks, *p* = 0.89; adjusted hits at 12 weeks, *p* = 0.98; unadjusted hits at 52 weeks, *p* = 0.94; adjusted hits at 52 weeks, *p* = 0.84; Table 4).

**Table 4.** Ratio of total hits in the antidepressant discontinuation group, relative to long-term maintenance treatment, 12 and 52 weeks after randomisation

Effect of treatment allocation				Interaction between treatment allocation and word valence			
Model	*n*	Hits ratio (95% CI)	*p* value	Model	*n*	Hits ratio (95% CI)	*p* value
12 weeks							
Unadjusted	433	1.03 (0.93–1.15)	0.56	Unadjusted	433	1.01 (0.85–1.20)	0.89
Adjusted^a^	430	1.00 (0.91–1.09)	0.92	Adjusted^a^	430	1.00 (0.84–1.18)	0.98
52 weeks							
Unadjusted	375	1.06 (0.94–1.18)	0.35	Unadjusted	375	0.99 (0.82–1.20)	0.94
Adjusted^a^	368	1.02 (0.93–1.12)	0.70	Adjusted^a^	368	0.98 (0.81–1.19)	0.84

Note. CI, confidence interval.

a Adjusted for baseline positive and negative hits, positive and negative false alarms, and stratification variables (symptom severity at baseline, assessed using the CIS-R, medication and study centre). Results unaltered after adjusting for predictors of missingness.

Findings were unaltered when including predictors of missingness or after limiting the sample to participants who adhered to study medication or those who performed well on the ECAT (online Supplement).

### Self-referential recall and risk of relapse

Over the 52-week follow-up, 39% of patients in the maintenance group and 56% in the discontinuation group relapsed (Lewis et al., 2021). Relapse rates were similar across all positive and negative recall scores.

There was no evidence of an association between positive word recall 12 weeks after randomisation and subsequent relapse (unadjusted hazard ratio = 0.97, CI 0.89–1.06, *p* = 0.55; fully adjusted hazard ratio = 0.99, CI 0.89–1.09, *p* = 0.81), and no evidence of an association between negative word recall 12 weeks after randomisation and subsequent relapse (unadjusted hazard ratio = 0.97, CI 0.86–1.10, *p* = 0.66; fully adjusted hazard ratio = 0.98, CI 0.84–1.14, *p* = 0.78; Table 5).

**Table 5.** Associations between the number of positive and negative words correctly recalled at baseline or 12 weeks after randomisation and time to first depression relapse

Recall at baseline and time to first depression relapse				Recall at 12 weeks and time to first depression relapse		
Model	*n*	Hazard ratio (95% CI)	*p* value	*n*	Hazard ratio (95% CI)	*p* value
Model 1: Association with positive hits						
Unadjusted	425	1.03 (0.95–1.10)	0.52	331	0.97 (0.89–1.06)	0.55
Partially adjusted^a^	425	1.03 (0.94–1.12)	0.54	331	0.99 (0.90–1.09)	0.87
Fully adjusted^b^	421	1.02 (0.93–1.12)	0.74	329	0.99 (0.89–1.09)	0.81
Model 2: Association with negative hits						
Unadjusted	425	1.03 (0.94–1.13)	0.50	331	0.97 (0.86–1.10)	0.66
Partially adjusted^a^	425	1.01 (0.91–1.12)	0.86	331	1.00 (0.87–1.16)	0.95
Fully adjusted^b^	421	1.01 (0.90–1.13)	0.87	329	0.98 (0.84–1.14)	0.78

Note. CI, confidence interval.

a Positive hits adjusted for negative hits and (positive and negative) false alarms. Negative hits adjusted for positive hits and (positive and negative) false alarms.

b Partially adjusted model (*) further adjusted for treatment allocation, medication, symptom severity at baseline, previous episodes of depression, duration of treatment prior to randomisation, sex, age, education. Results unaltered after adjusting for predictors of missingness.

We also found no evidence of an association between baseline word recall and relapse, independent of treatment allocation (Table 5). This was the case for both positive recall (unadjusted hazard ratio = 1.03, CI 0.95–1.10, *p* = 0.52; fully adjusted hazard ratio = 1.02, CI 0.93–1.12, *p* = 0.74) and negative recall (unadjusted hazard ratio = 1.03, CI 0.94–1.13, *p* = 0.50; fully adjusted hazard ratio = 1.01, CI 0.90–1.13, *p* = 0.87). Hazard ratios greater than 1 indicate that (positive or negative) word recall at baseline was positively associated with relapse (bad prognosis).

These findings were unaltered when restricting analyses to participants who performed well on the ECAT at baseline (online Supplement). Further analyses exploring the interaction between recall at baseline and treatment effects are included in the online Supplement (Tables S4 and S5).

## Discussion

In a large sample of primary care patients, we found no evidence that discontinuing long-term maintenance antidepressant treatment affected positive or negative self-referential recall. We also found no evidence that self-referential recall prior to or following antidepressant discontinuation was associated with risk of relapse, independent of treatment. Changes in emotional memory therefore do not appear to be a valid marker of antidepressant discontinuation nor a useful predictor of depression relapse.

### Integration with existing findings

Our findings are not consistent with previous studies showing an association between antidepressant drug action and emotional recall (Arnone et al., 2009; Harmer et al., 2004, 2009a), and they do not support the hypothesis that altered emotional processing, as assessed by emotional recall, is a marker of relapse risk (Bouhuys et al., 1999). These studies were based on small samples, often with healthy individuals and mostly acute or short-term interventions, which may have limited their statistical power and generalisability to primary care patients.

Previous studies have not consistently supported the notion of self-referential recall as a marker of antidepressant action (Komulainen et al., 2018; Walsh et al., 2018). More recently, a large placebo-controlled trial of primary-care patients with depressive symptoms found no evidence that the SSRI sertraline altered positive or negative recall early in treatment (Ahmed et al., 2021). Together with the present study, these findings challenge the neuropsychological model of antidepressant action, at least in relation to recall of positive and negative words; it is possible that tasks assessing other emotional and cognitive processes would be more sensitive to the influence of antidepressants. They also highlight the importance of conducting large, high-quality trials that can generalise to primary-care patients, where most depression is treated, when investigating the effects of antidepressant treatment on emotional processing.

### Strengths and limitations

The ANTLER trial was the largest individual trial of long-term maintenance antidepressant treatment not funded by the pharmaceutical industry. While other studies have investigated antidepressant discontinuation following short-term treatment for an acute depressive episode, over 70% of ANTLER participants had been receiving medication for more than 3 years (Lewis et al., 2021). We expect our findings to be generalisable to the population who currently receive maintenance treatment within primary care and are well enough to consider stopping, although they may not translate beyond long-term depression samples.

All participants were primary care patients currently taking one of four of the most commonly prescribed antidepressants for first-line treatment. The medications investigated comprise 75% of all long-term antidepressant prescriptions in England (Duffy et al., 2019). Given that changes in emotional processing are thought to be a common mechanism of action of all antidepressant agents, our findings should apply to other classes of antidepressants (Harmer et al., 2017).

Our study sample was large, pragmatic, and participants were randomised, reducing the risk of Type II (false negative) errors and confounding. However, this may have introduced larger measurement errors, potentially making it more difficult to detect small changes in emotional processing compared with experimental research settings. Given that randomisation was not taken into account when investigating the association between recall and risk of relapse, residual confounding cannot be excluded from this part of the analysis.

Participants performed the word recall task three times over the course of the trial. Although a different set of words was presented at each time point, participants would have expected the free recall test at 12 and 52 weeks, so the recall element of the task was only a surprise at baseline. Nevertheless, consistent with previous findings, we did not observe an improvement in recall over time, suggesting that awareness of the recall test is insufficient to alter emotional memory (Ahmed et al., 2021; Lewis et al., 2017).

The task was only administered at baseline and 12 and 52 weeks after randomisation. At baseline, it is likely that emotional processing biases may have been masked by antidepressant treatment. Due to tapering, participants in the discontinuation group had been medication-free for four weeks by the first follow-up. Antidepressants are thought to alter emotional processing very early in treatment, so changes in emotional processing might have occurred before the 12-week follow-up. This may have limited our ability to predict relapse. However, it is unclear why group differences in self-referential recall would no longer be present by week 12, especially considering that differences in depressive symptoms were highest at this time and that these findings were unaltered after adjusting for adherence to study medication (Lewis et al., 2021).

## Conclusion

Despite the substantial body of research demonstrating associations between emotional memory and antidepressant agents, we did not find evidence that discontinuing long-term maintenance antidepressant treatment affected self-referential recall or that self-referential recall was associated with risk of relapse. The effects of antidepressant discontinuation on other forms of emotional processing have not been comprehensively addressed.

## Supplementary material

For supplementary material accompanying this paper visit https://doi.org/10.1017/S0033291722003981. click here to view supplementary material
