# Supplementary material for: Neuropsychological markers of antidepressant action: a secondary analysis of the ANTLER randomised controlled trial
Source: Psychol Med. 2023 Feb 2;53(14):6592–9. doi: 10.1017/S0033291722003981 (PMC10600933; doi:10.1017/S0033291722003981)
Supplement: Supplementary file 1 [file S0033291722003981sup001.pdf]

SUPPLEMENTARY MATERIAL

Methods

The main analysis was repeated including only participants who scored 80% or above on the ECAT at baseline and adhered to study medication.

Performance on the ECAT was calculated as the proportion of personality characteristics classified accurately ('like' for positive words and 'dislike' for negative words). Only participants with a score of 80% or over at baseline were included in the analysis.

Adherence to study medication was assessed at 12 and 52 weeks using a five-item self-report measure of compliance (Tallon et al., 2016). Only participants who adhered to study medication were included in analyses of the association between antidepressant discontinuation and recall.

Further analyses investigated potential interactions between the effect of antidepressant discontinuation on relapse risk and recall at baseline using Cox Proportional Hazards modelling. The outcome was time to relapse, and we included an interaction term between positive or negative hits at baseline and treatment allocation.

Models were adjusted for treatment allocation, medication, and potential confounders. A stratified analysis for low (below the median) and high (equal to or above the median) baseline hits was performed to illustrate interaction effects. These analyses were repeated including only participants who scored 80% or above on the ECAT at baseline.

Results

Emotional categorisation task (ECAT)

Baseline ECAT data were available for 434 participants: 225 in the maintenance and 209 in the discontinuation group (data were missing for n = 3 participants due to technical issues).

Performance in the ECAT was good at baseline (median percent correct = 92.5%, IQR = 10%) and at 12 and 52 weeks (median percent correct = 95%, IQR = 7.5%), with no differences between antidepressant maintenance and discontinuation.

Some participants failed to understand some of the words (14% scored below 80% at baseline), and 4% performed worse than chance.

Antidepressant discontinuation and self-referential recall

The results of the main analysis were unaltered after adjusting for performance on the ECAT and adherence to study medication. There was no evidence of an association between treatment allocation and positive hits, negative hits or total hits, and no interaction between treatment allocation and word valence (Tables S1 and S2).

Self-referential recall at baseline and risk of relapse

Restricting analyses to participants who scored 80% or above on the ECAT at baseline did not affect our findings on the lack of an association between recall at baseline and risk of relapse. Positive and

negative hits at baseline failed to predict relapse, both before and after adjustment for treatment and potential confounding factors (Table S3).

Effect modification by self-referential recall

There was evidence of an effect modification by baseline negative recall (adjusted hazard ratio = 0.79, CI = 0.65-0.96, p = 0.02), suggesting that individuals with lower negative recall may benefit the most from maintenance antidepressants as a relapse prevention strategy (Table S4).

Consistent with this, the risk of relapse associated with discontinuation was higher for participants with low baseline negative hit scores (adjusted hazard ratio = 3.15, CI = 1.88-5.27, p < 0.001) than for those with high scores (adjusted hazard ratio = 1.66, CI = 1.15-2.41, p = 0.01).

We found no evidence of an effect modification by positive hits (Table S4).

These subgroup analyses had reduced statistical power and should be interpreted with caution. The baseline negative hits by group interaction effect was no longer significant when we excluded participants who scored below 80% on the ECAT (Table S5).

References

Tallon, D., Wiles, N., Campbell, J., Chew-Graham, C., Dickens, C., Macleod, U., ..., Kessler, D. (2016). Mirtazapine added to selective serotonin reuptake inhibitors for treatment-resistant depression in primary care (MIR trial): study protocol for a randomised controlled trial. *Trials*, 17(1), 1-14. https://doi.org/10.1186/s13063-016-1199-2

Table S1. Ratio of positive or negative hits in the antidepressant discontinuation group, relative to long-term maintenance treatment, 12 and 52 weeks after randomisation, restricted to participants who adhered to study medication and performed at 80% accuracy or above on the baseline ECAT.

Positive hits				Negative hits			
**Model**	**n**	**Hits ratio (95% CI)**	**p value**	**Model**	**n**	**Hits ratio (95% CI)**	**p value**
**12 weeks**							
**Unadjusted**	341	1.04 (0.91 to 1.20)	0.57	**Unadjusted**	341	1.01 (0.87 to 1.19)	0.85
**Adjusted***	338	1.04 (0.92 to 1.18)	0.52	**Adjusted***	338	0.98 (0.84 to 1.14)	0.75
**52 weeks**							
**Unadjusted**	198	1.04 (0.87 to 1.24)	0.70	**Unadjusted**	198	1.02 (0.81 to 1.27)	0.87
**Adjusted***	196	1.00 (0.83 to 1.20)	0.99	**Adjusted***	196	0.97 (0.79 to 1.19)	0.77

Note. CI, confidence interval. *Positive hits adjusted for negative hits, baseline positive hits and stratification variables (symptom severity at baseline, assessed using the CIS-R, medication and study centre). Negative hits adjusted for positive hits, baseline negative hits and stratification variables. Results unaltered after adjusting for predictors of missingness.

Table S2. Ratio of total hits in the antidepressant discontinuation, relative to long-term maintenance treatment, 12 and 52 weeks after randomisation, restricted to participants who adhered to study medication and performed at 80% accuracy or above on the baseline ECAT.

Effect of treatment allocation				Interaction between treatment allocation and word valence			
**Model**	**n**	**Hits ratio (95% CI)**	**p value**	**Model**	**n**	**Hits ratio (95% CI)**	**p value**
**12 weeks**							
**Unadjusted**	341	1.02 (0.91 to 1.15)	0.72	**Unadjusted**	341	0.97 (0.81 to 1.18)	0.79
**Adjusted***	338	0.99 (0.90 to 1.10)	0.92	**Adjusted***	338	0.96 (0.79 to 1.16)	0.65
**52 weeks**							
**Unadjusted**	198	1.02 (0.87 to 1.20)	0.77	**Unadjusted**	198	0.98 (0.75 to 1.28)	0.90
**Adjusted***	196	0.95 (0.83 to 1.10)	0.48	**Adjusted***	196	0.98 (0.76 to 1.28)	0.89

Note. CI, confidence interval. *Adjusted for baseline positive and negative hits and stratification variables (symptom severity at baseline, assessed using the CIS-R, medication and study centre). Results unaltered after adjusting for predictors of missingness.

Table S3. Associations between the number of positive and negative words correctly recalled at baseline and time to first depression relapse in participants who performed at 80% accuracy or above on the baseline ECAT.

Time to first depression relapse			
**Model**	**n**	**Hazard ratio (95% CI)**	**p value**
**Model 1: Association with positive hits**			
**Unadjusted**	366	1.02 (0.94 to 1.11)	0.67
**Partially adjusted***	366	1.05 (0.96 to 1.15)	0.32
**Fully adjusted†**	362	1.02 (0.92 to 1.12)	0.73
**Model 2: Association with negative hits**			
**Unadjusted**	366	0.99 (0.90 to 1.10)	0.90
**Partially adjusted***	366	0.96 (0.86 to 1.07)	0.48
**Fully adjusted†**	362	0.98 (0.87 to 1.10)	0.73

Note. CI, confidence interval. *Positive hits adjusted for baseline negative hits. Negative hits adjusted for baseline positive hits. †Partially adjusted model (*) further adjusted for treatment allocation, medication, symptom severity at baseline, previous episodes of depression, duration of treatment prior to randomisation, sex, age, education. Results unaltered after adjusting for predictors of missingness.

Table S4. Effect modification of the association between antidepressant discontinuation and time to first depression relapse by baseline positive and negative hits.

Time to first depression relapse			
**Model**	**n**	**Hazard ratio (95% CI)**	**p value**
**Model 1: Modification by baseline positive hits**			
**Unadjusted**	425	1.01 (0.86 to 1.19)	0.91
**Partially adjusted***	425	1.01 (0.85 to 1.19)	0.93
**Fully adjusted†**	421	1.00 (0.84 to 1.19)	0.97
**Model 2: Modification by baseline negative hits**			
**Unadjusted**	425	0.80 (0.66 to 0.96)	0.02
**Partially adjusted***	425	0.80 (0.66 to 0.97)	0.02
**Fully adjusted†**	421	0.79 (0.65 to 0.96)	0.02

Note. CI, confidence interval. *Positive hits adjusted for baseline negative hits. Negative hits adjusted for baseline positive hits. †Partially adjusted model (*) further adjusted for treatment allocation, medication, symptom severity at baseline, previous episodes of depression, duration of treatment prior to randomisation, sex, age, education.

Table S5. Effect modification of the association between antidepressant discontinuation and time to first depression relapse by baseline positive and negative hits in participants who performed at 80% accuracy or above on the ECAT.

Time to first depression relapse			
**Model**	**n**	**Hazard ratio (95% CI)**	**p value**
**Model 1: Effect modification by baseline positive hits**			
**Unadjusted**	366	1.08 (0.91 to 1.28)	0.40
**Partially adjusted***	366	1.06 (0.89 to 1.27)	0.52
**Fully adjusted†**	362	1.07 (0.88 to 1.29)	0.50
**Model 2: Effect modification by baseline negative hits**			
**Unadjusted**	366	0.86 (0.70 to 1.05)	0.13
**Partially adjusted***	366	0.86 (0.71 to 1.05)	0.15
**Fully adjusted†**	362	0.87 (0.70 to 1.07)	0.18

Note. CI, confidence interval. *Positive hits adjusted for baseline negative hits. Negative hits adjusted for baseline positive hits. †Partially adjusted model (*) further adjusted for treatment allocation, medication, symptom severity at baseline, previous episodes of depression, duration of treatment prior to randomisation, sex, age, education.
